# Clinical importance of ADC in the prediction of ^125^I in the treatment for gliomas

**DOI:** 10.7150/jca.50789

**Published:** 2021-01-30

**Authors:** Congxiao Wang, Zhijian Xu, Song Wang, Lijing Peng, Wei Zhang, Xueda Li, Lili Yang, Ying Luan, Tao Su, Zixiang Li, Xiaokun Hu

**Affiliations:** 1 Department of the Interventional Medical Center, the Affiliated Hospital of Qingdao University, Qingdao 266000, Shandong, China.; 2 Department of Clinical Laboratory, the Affiliated Hospital of Qingdao University, Qingdao 266000, Shandong, China.; 3 JinHua Municipal Central Hospital, JinHua, 321000, Zhejiang, China.; 4 Jiangsu Key Laboratory of Molecular and Functional Imaging, Department of Radiology, Zhongda Hospital, Medical School of Southeast University, Nanjing, 210009, China.

**Keywords:** Gliomas, minimum ADC, ^125^I brachytherapy

## Abstract

**Objectives:** To determine whether the minimum apparent diffusion coefficient (minADC) value can stratify survival in patients with glioma before ^125^I brachytherapy.

**Methods:** The study was approved by the Institutional Review Board, and the requirement for informed consent was waived. Twenty-three patients (16 male, 7 female; median age, 48 years) with high-grade glioma (HGG) (n=9) or recurrence after multimodal treatment (n=14) were included in this study. minADC values were obtained before ^125^I implantation. Overall survival (OS) and progression-free survival (PFS) were analyzed with Cox proportional hazards regression models and the Kaplan-Meier method with the log-rank test.

**Results:** For ^125^I-treated patients, the hazard ratio for OS in patients with ADC≥1.0*10^^-3^ mm^2^·sec^-1^ (high minADC) versus ADC<1.0*10^^-3^ mm^2^·sec^-1^ (low minADC) was 0.220 (95% confidence interval: 0.066, 0.735). The median OS was 12 months for patients with high minADC values and 6.0 months for those with low minADC values, and the differences were significant (p=0.032). The median PFS was 12 months for patients with high minADC values and 4 months for those with low minADC values. Significant differences were found in the long-rank test (p=0.013). The multivariate analysis results showed that minADC pre-^125^I implantation was an independent predictor of OS and PFS in patients receiving ^125^I brachytherapy.

**Conclusions:** Pre-^125^I implantation ADC analysis can stratify prognosis in ^125^I-treated patients with glioma, which may aid in choosing a suitable therapy for glioma patients.

## Introduction

Malignant primary brain tumors, 80% of which are gliomas, account for a large number of deaths worldwide 1. There are four different grades of gliomas (grade I-IV) according to the WHO criteria, and grade III and IV gliomas are also called high-grade or malignant gliomas 2. The prognosis of high-grade gliomas (HGGs) is poor regardless of the variety of therapies. Gliomas easily recur, and even gross total resection has been performed following adjuvant radiotherapy and chemotherapy due to its characteristic of diffusely infiltrating spread from the origin site 3, 4.

^125^I brachytherapy has been suggested to improve the survival of patients with primary or recurrent glioma as an initial or a salvage therapy method in previous studies 5-8. However, in the clinical setting, the prognosis of patients with glioma treated with ^125^I brachytherapy varies; some patients have a relatively favorable prognosis and some do not, even with the same histopathology and similar therapies. Diffusion-weighted imaging (DWI) shows additional information derived from the microscopic motion of water protons at the cellular or physiologic level in addition to what conventional MRI shows 9. ADC values could be obtained from DWI. Several studies reported inverse correlations between the apparent diffusion coefficient (ADC) and tumor cellularity as well as glioma grade, and DWI has been used for the assignment of tumor grades or the differentiation of tumors 10-12*.* A low ADC value reflects high cell density because most of the water molecules are restricted to move within cells rather than in the extracellular space 13. Thus, ADC values have been applied for the assessment of therapy response in brain tumors and for the prediction of survival 14,15. However, to the best of our knowledge, no studies have been reported on whether ADC could be used to assess the glioma response to ^125^I brachytherapy. Thus, the purpose of our study was to retrospectively evaluate whether pre-^125^I implantation ADC analysis has prognostic value in patients with glioma undergoing ^125^I brachytherapy.

## Materials and Methods

### Patient criteria

This retrospective study was approved by the institutional review board, and the requirement for informed patient consent was waived. The study spanned from March 4, 2014, to November 11, 2019. Patients who met the following inclusion criteria were included in our study: (a) patients with primary HGG or recurrent glioma; (b) patients who received ^125^I implantation; (c) patients with diffusion-weighted images obtained within 1 month before ^125^I implantation; and (d) patients with no previous or concurrent malignant diseases. Patients were excluded due to (a) the unavailability of digital pretreatment MRI data or (b) the presence of intratumoral hemorrhage.

### Treatment

Two interventional radiologists who were both blinded to the research reviewed the patients' medical records to obtain the therapy information. Patients with recurrent glioma (n=14) underwent resection of the tumor at the time of the first diagnosis. Eleven of the patients underwent postoperative external-beam radiation therapy with radiation from 30 Gy to 64.2 Gy and underwent adjuvant chemotherapy with temozolomide (TMZ) using the Stupp protocol. Nine patients received ^125^I brachytherapy as their initial treatment due to extensive lesions, poor medical conditions, or the rejection of surgery and adjuvant radiochemotherapy. The ^125^I implantation was performed by one interventional radiologist with 17 years of experience. All patients underwent outpatient examinations monthly, and head MRI or CT was carried out almost every 2 months.

### MR imaging and data processing

Pretreatment MRI was performed with a 3.0 Tesla scanner, including T1-weighted imaging (TSE sequence, field of view = 25 cm ×25 cm, slice thickness = 5 mm, interslice distance = 1.5 mm, matrix size=256 mm × 204 mm, TR = 1800 ms, TE =8.5 ms), T2-weighted imaging (TSE sequence, field of view = 25 cm × 25 cm, slice thickness = 5 mm, interslice distance = 1.5 mm, matrix size=320 mm × 296 mm, TR = 3800 ms, TE =92 ms), diffusion-weighted imaging (EPI sequence, field of view = 25 cm × 25 cm, slice thickness = 5 mm, interslice distance =1.5 mm, matrix size = 19.2 cm × 19.2 cm, TR = 6300 ms, TE=95 ms), and contrast material-enhanced T1-weighted imaging (gadopentetate dimeglumine, Magnevist; Berlex Laboratories, Wayne, NJ) (TSE sequence, field of view = 25 cm × 25 cm, slice thickness = 5 mm, interslice distance = 1.5 mm, TR = 1800 ms, TE=8.5 ms, matrix size = 256 × 204). ADCs were calculated with b=0 s/mm^2^ and b=1000 s/mm^2^. Postcontrast images were acquired immediately after contrast agent injection.

ADC maps were constructed via software installed on the MR unit. Two radiologists with 17 and 10 years of experience who were both blinded to the research identified the solid tumor components with or without enhancement on the MRI images in consensus. Five to ten regions of interest (ROIs) were manually placed within the solid tumor component on the ADC map by the above two radiologists. No cystic or necrotic areas or skull, which may influence the ADC values, were allowed to cover the ROI. minADC values were obtained for further analysis.

### Study outcomes

The outcomes observed were the median overall survival (OS) and median progression-free survival (PFS). Overall survival was measured from the time of ^125^I implantation to the time of death or the last follow-up. Progression-free survival was measured from the time of ^125^I implantation to the time of tumor recurrence or progression or death.

### Iodine-125 implantation

**Figure 1:**
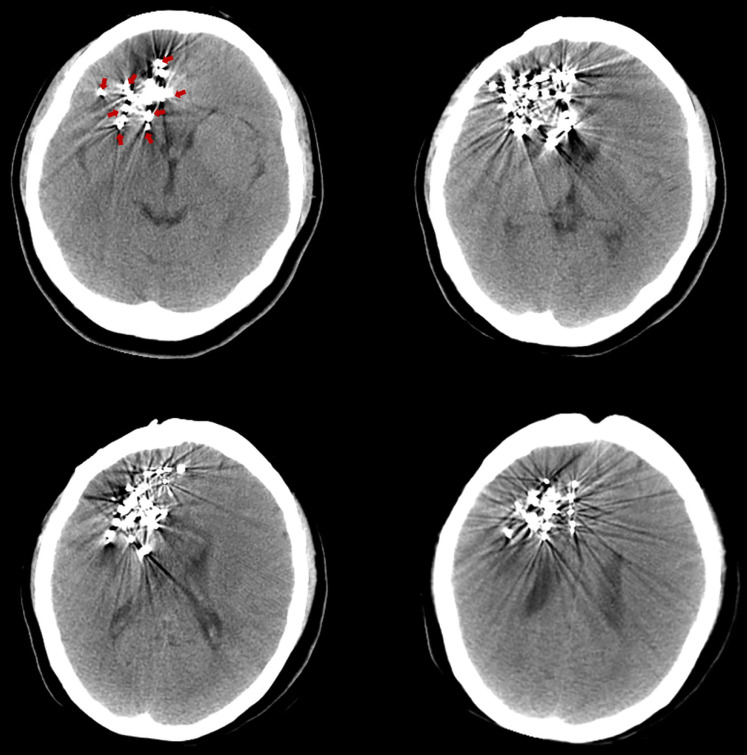
Three days after the ^125^I implantation, a CT scan was carried out to exclude delayed damage to the brain. The images show different slices of the brain with ^125^I seeds. (Arrows indicate the ^125^I seeds implanted in the brain).

The pre-^125^I implantation plan was performed with the computerized treatment planning system (TPS, Beijing Astro Technology Ltd., Co., Beijing, China). With a negative pressure vacuum pad, the patients were fixed on the CT scan bed. After anesthetization with 2% lidocaine, an incision of approximately 2 mm was made on the scalp. Then, 2-4 mm diameter holes on the skull were made with an electric cranial drill. Flat needles were inserted into the tumor, and the angle and depth of the needle were dynamically adjusted under CT guidance. Iodine-125 seeds (diameter of 0.8 mm, length of 4.5 mm, half-life of 59.4 days; Model 7711, Beijing Atom and High Technique Industries, Inc., Beijing, China) were implanted, and dosimetric verification was performed with TPS during the operation. The scalp was sutured post-^125^I implantation to avoid leakage of the cerebrospinal fluid. After the implantation, vital signs were monitored, and the patients were required to be inactive on the bed for 24 hours. Routine dehydration medications were given for 7 to 14 days. Three days after the ^125^I implantation, a CT scan was carried out to exclude delayed damage to the brain (Fig. 1).

### Statistical analysis

The last follow-up date was April 22, 2020. The data were analyzed with SPSS (Version 18.0, IBM, NY, USA). A Cox proportional hazards regression model was used for the univariate analysis and all variables with P≤0.1 or variables that were clinically valuable were included in the multivariate analysis using a Cox proportional hazards regression model. Survival curves were calculated with the Kaplan-Meier method, and the differences were analyzed with the log-rank test. For all analyses, P <0.05 was considered to indicate a significant difference.

## Results

### Patient characteristics

**Figure 2:**
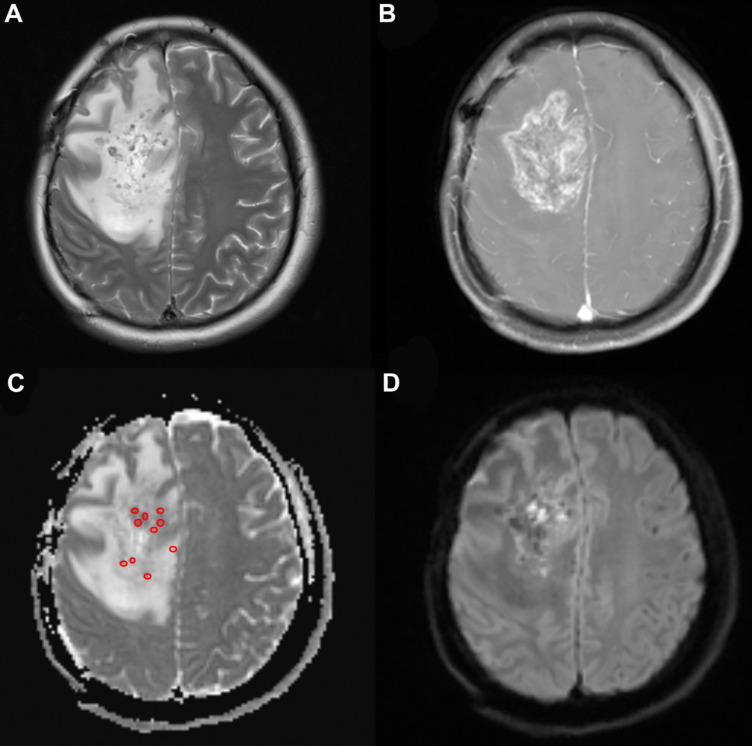
A 33-year-old woman with high-grade glioma (WHO grade IV). (a) Pre-^125^I implantation T2WI, (b) contrast-enhanced T1WI, (c) ADC map (circles indicate the ADC values measured on the ADC map), and (d) DWI showing the glioma location in the brain. The minimum ADC value was detected on the ADC map.

Table 1 shows the baseline characteristics of the patients with primary and recurrent glioma. Twenty-three patients (16 male and 7 female) were included in the present study. Four patients were over 65 years old. The median KPS (Karnofsky performance score) was 80 (range, 50-90). Of the patients with recurrent glioma, 12 underwent gross total resection and 2 underwent partial resection before. Eleven of the patients received radiotherapy with a total median radiation dose of 60 Gy (range: 30-64.2 Gy) and chemotherapy with temozolomide (TMZ) using the Stupp protocol. There were 3 patients who rejected radiochemotherapy after resection. Nine patients with primary glioma had MRI- and CT-proven high-grade glioma and received ^125^I brachytherapy as their initial treatment rather than conventional therapy with their informed consent due to extensive lesions, poor medical conditions or the rejection of surgery and adjuvant radiochemotherapy. Biopsy was performed during the ^125^I implantation to confirm the diagnosis. Minimum ADC values were obtained from the ADC map (Fig. 2). The mean minADC value was 0.96. The number of patients with tumors located in the left hemisphere, right hemisphere, both hemispheres and the cerebellum was 10, 9, 3, and 1, respectively. The median volume of the tumor was 37.33 cm^3^. Aggravating brain edema was found in seven of these patients, which was relieved after dehydration treatment approximately 15 days later. No severe postoperative complications were found in these patients.

### Survival analysis

**Figure 3:**
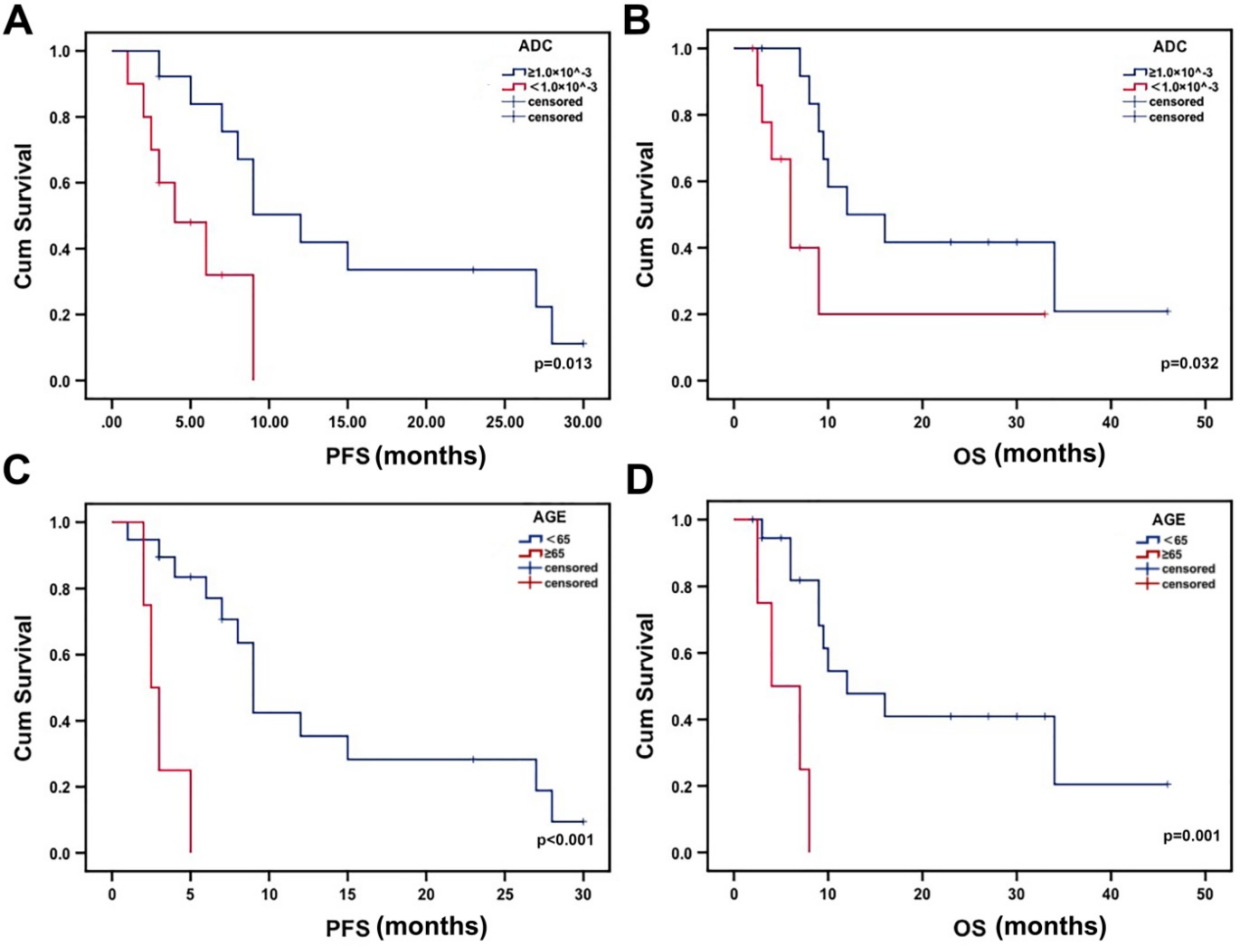
Kaplan-Meier estimates of OS and PFS from the time of ^125^I implantation. There were significant differences in PFS and OS according to minADC (cut-off value: 1.00×10^^-3^ mm^2^ sec^-1^) and age.

The median PFS was 4 months for patients with low minADC values and 12 months for those with high minADC values (p=0.013, Fig. 3a). The median OS was 6 months for patients with low minADC values and 12 months for those with high minADC values. The differences between patients with low and high minADC values were statistically significant (p=0.032, Fig. 3b). The median PFS was 9 months in the younger group (age <65) and 2.5 months in the older group (age ≥65) (p<0.001, Fig. 3c). The median OS was 12 months in the younger group and 4 months in the older group, and the difference was significant (p=0.001, Fig. 3d). As shown in Table 2, in the univariate Cox model, age, minADC, and KPS were the most accurate predictors of OS (age: OR=9.028, p=0.004, 95% CI: 1.990, 40.968; minADC: OR=3.256, p=0.042, 95% CI: 1.041, 10.186; KPS: OR=0.299, p=0.038, 95% CI: 0.096, 0.935) and PFS (age: OR=10.878, p=0.002, 95% CI: 2.347, 50.426; minADC: OR=3.861, p=0.022, 95% CI: 1.214, 12.275; KPS: OR=0.148, p=0.020, 95% CI: 0.029, 0.744). When these factors were included in the multivariate Cox model, age and minADC were still the most accurate predictors of OS (age: HR=13.438, p=0.002, 95% CI: 2.655, 68.010; minADC: HR=0.220, p=0.014, 95% CI: 0.066, 0.735) and PFS (age: HR=42.533, p<0.001, 95% CI: 5.254, 344.303; minADC: HR=0.164, p=0.008, 95% CI: 0.043, 0.624) (Table 3).

## Discussion

Our results indicated that pre-^125^I implantation ADC analysis was valuable for assessing ^125^I treatment for HGG. HGG is a disease that mainly grows from the site of origin and can spread throughout the whole brain. Due to its diffusely infiltrating characteristics, gliomas can invade the adjacent brain for some distance, and they are invisible to clinical examination, preventing complete gross resection and promoting resistance to radiochemotherapy 16. As a result, relapses of HGG are always a difficult subject for doctors, regardless of the multiple conventional therapies available 17, 18. In addition, facing recurrent glioma, which therapy methods should be chosen remains a question.

In patients with glioma, ^125^I showed therapeutic efficacy, regardless of primary glioma or recurrent glioma 7, 8, 19. However, not all patients respond well to ^125^I treatment. Therefore, research on biomarkers that predict the outcomes of ^125^I treatment is necessary for clinical treatment, which may aid in the selection of a suitable therapy for glioma patients. ADC reveals the microscopic structure of the tumor, which implies necrotic cell clusters and cell density at the cellular or physiological level that offer indirect information about tumor aggressiveness 20. Previous studies confirmed the correlation of ADC with the prognosis of the tumor. Yamasaki et al.'s results showed an inverse relationship between ADC and astrocytic tumors 21. Ryuji Murakami's research confirmed the value of ADC in the prediction of postoperative radiation therapy response in malignant gliomas. In another study, Wu CC et al indicated that lower ADC values correlated with poor survival in patients, regardless of WHO grade 22. Although many studies have confirmed the predictive value of pretreatment ADC, iodine-125 seeds could play continuous roles within the tumor, with the continuous release of low-dose γ rays, which is different from EBRT. Thus, whether ADC analysis could be used for the prediction of ^125^I efficacy is unknown. In the present study, we hypothesized that pre-^125^I implantation ADC could be a prognostic predictor for ^125^I treatment for gliomas.

In our study, patients with primary or recurrent glioma were included in the analysis, and DWI was carried out within 1 month before the implantation of ^125^I. The cut-off value of 1.00×10^^-3^ mm^2^ sec^-1^ was chosen as previously reported 22. As the univariate analysis results showed, age, KPS, and minADC were predictors of both OS and PFS. After the multivariate analysis, age and minADC were independent predictors of OS and PFS. However, KPS showed no effects on OS and PFS. These results indicated that younger patients with a minADC value ≥1.00×10^^-3^ mm^2^ sec^-1^ have a better prognosis with ^125^I brachytherapy. Previous studies suggested that KPS and age were independent predictors of OS in patients with glioma following resective surgery, which was partly in line with the results in our study 17. Interestingly, the present study showed that the pre-^125^I implantation minADC was an important predictor of the prognosis of glioma patients receiving ^125^I brachytherapy, with p-values of 0.014 and 0.008 for OS and PFS, respectively, which has not been reported in previous studies to the best of our knowledge.

The results of the Kaplan-Meier analysis with the log-rank test showed significant differences in OS and PFS between patients with low and high minADC values. Additionally, differences were found between different age groups. The results indicated that low minADC values correlated with poor survival in patients receiving ^125^I treatment. Thus, the cut-off value of 1.00×10^-3 mm^2^ sec^-1^ could be used to evaluate the prognosis of patients before ^125^I implantation. The results confirmed the importance of pre-^125^I implantation ADC analysis, which could be used to evaluate the prognosis of patients receiving ^125^I implantation. In addition, our results also indicated that young patients receiving ^125^I brachytherapy for glioma had a better prognosis. ADC values were easy to obtain with the construction of the DWI image, allowing the evaluation of the different therapy responses to ^125^I implantation. Pre-^125^I implantation ADC analysis is an important and convenient method, helping patients with glioma select treatment options.

Nine patients received ^125^I seeds as their initial therapy method instead of the standard therapy due to extensive lesions, poor medical conditions, or the rejection of surgery and adjuvant radiochemotherapy. Patients with primary glioma underwent ^125^I brachytherapy as a salvage therapy method because they were not suitable for the conventional therapy methods, which may contribute to the results that no significant differences were found in OS and PFS between primary and recurrent gliomas. The greatest limitation of the study was that the sample size was not large enough; however, we still believe that the study offered valuable insights for clinical treatment with ^125^I brachytherapy. More patients with glioma who received ^125^I treatment will be included in future research.

We already carried out more than 600 cases of ^125^I implantation in the brain. Most operations were carried out under local anesthesia, which is tolerable. Although the implantation of ^125^I seeds was carried out with an 18G flat needle, which caused minor damage to the brain, the puncturing operation sometimes caused aggravating brain edema and bleeding of the tumor. The condition of patients with aggravating brain edema improved a few months later with dehydration treatment, and self-absorption of the cerebral hemorrhage was almost completed approximately 15 days later with the conservation treatment. Once heavy brain edema and brain bleeding happened after ^125^I implantation, which may cause cerebral hernia, a surgical operation will be carried out.

In conclusion, the present study showed that patients with high minADC values (<1.00×10^-3 mm^2^ sec^-1^) had better outcomes with ^125^I brachytherapy. Pretreatment minADC values supplied valuable information for the prognosis of ^125^I treatment, which had not been reported to the best of our knowledge and may supply an important and convenient method for therapy decision making.

## Key points

Pre-^125^I implantation ADC analysis can stratify prognosis in ^125^I-treated patients with gliomas.

Patients with lower minADC values had a poor prognosis receiving ^125^I brachytherapy for gliomas.

## Figures and Tables

**Table 1:** Baseline characteristics of the patients

Parameters	Values
Age (years)	
**Median (range)**	48 (16-70)
≥65	4
<65	19
**Sex**	
Male	16
Female	7
KPS	
**Median (range)**	80 (50-90)
≥80	12
<80	11
**Resection**	
Gross Total Resection	12
Partial Resection	2
Biopsy	9
**EBRT**	
Yes	11
No	12
**Adjuvant Chemotherapy**	
Yes	11
No	12
**Minimum ADC (mm^2^.sec^-1^)**	
Mean±SD	(0.96±0.32)×10^^-3^
≥1.0×10^^-3^	13
<1.0×10^^-3^	10
**Tumor Location**	
Left Hemisphere	10
Right Hemisphere	9
Both Hemispheres	3
Cerebellum	1
**Tumor Type**	
Recurrent	14
Primary (high-grade)	9
**Tumor Volume (cm^3^)**	
Median (range)	37.44 (0.90-164.63)

Note. KPS, Karnofsky performance score; EBRT, external beam radiotherapy; ADC, apparent diffusion coefficient.

**Table 2:** Univariate analysis of OS and PFS in ^125^I-treated patients

Variables	Odds Ratio	*p* value	95% Confidence Interval
	**Overall Survival**		
Age	9.028	0.004	1.990, 40.968
Sex	1.351	0.621	0.411, 4.444
KPS	0.299	0.038	0.096, 0.935
Tumor Location	1.292	0.479	0.636, 2.627
Type	0.892	0.838	0.297, 2.675
Surgery	1.121	0.838	0.374, 3.364
EBRT	1.676	0.356	0.560, 5.015
Adjuvant Chemotherapy	1.676	0.356	0.560, 5.015
Minimum ADC	3.256	0.042	1.041,10.186
	**Progression-free Survival**		
Age	10.878	0.002	2.347, 50.426
Sex	1.503	0.431	0.545, 4.148
KPS	0.148	0.020	0.029, 0.744
Tumor Location	1.469	0.181	0.837, 2.580
Type	1.302	0.601	0.484, 3.505
Surgery	0.768	0.601	0.285, 2.068
EBRT	1.876	0.230	0.671, 5.244
Adjuvant Chemotherapy	1.876	0.230	0.671, 5.244
Minimum ADC	3.861	0.022	1.214, 12.275

**Table 3:** Multivariate Analysis of OS and PFS in ^125^I-treated patients

Variable	Hazard Ratio	*p* value	95% Confidence Interval
	**Overall Survival**		
Age	13.438	0.002	2.655,68.010
Minimum ADC	0.220	0.014	0.066,0.735
	**Progression-free Survival**		
Age	42.533	0.001	5.254,344.303
Minimum ADC	0.164	0.008	0.043,0.624

## Abbreviations

HGG: high-grade glioma
DWI: diffusion-weighted imaging
ADC: apparent diffusion coefficient
TMZ: Temozolomide
ROI: region of interest
KPS: Karnofsky performance score
TPS: treatment planning system
TSE: turbo spin-echo
EPI: Echo-planar imaging
